# Initial in vitro and in vivo assessment of Au@DTDTPA-RGD nanoparticles for Gd-MRI and 68Ga-PET dual modality imaging

**DOI:** 10.1186/2197-7364-2-S1-A89

**Published:** 2015-05-18

**Authors:** Charalmpos Tsoukalas, Gautier Laurent, Gloria Jiménez Sánchez, Theodoros Tsotakos, Rana Bazzi, Dimitris Stellas, Constantinos Anagnostopoulos, Lia Moulopoulos, Vasilis Koutoulidis, Maria Paravatou-Petsotas, Stavros Xanthopoulos, Stephane Roux, Penelope Bouziotis

**Affiliations:** National Center for Scientific Research "Demokritos", Greece; Université de Franche-Comté, Institut UTINAM, France; Biomedical Research Foundation, Academy of Athens, Greece; Department of Radiology, Areteion Hospital, University of Athens Medical School, Greece

Gadolinium chelate coated gold nanoparticles (Au@DTDTPA) can be applied as contrast agents for both in vivo X-ray and magnetic resonance imaging. In this work, our aim was to radiolabel and evaluate this gold nanoparticle with Ga-68, in order to produce a dual modality PET/MRI imaging probe. For a typical preparation of 68Ga-labeled nanoparticles, the Au@DTDTPA nanoparticles (Au@DTDTPA/Au@DTDTPA-RGD) were mixed with ammonium acetate buffer, pH 5 and 40 MBq of 68Ga eluate. The mixture was then incubated for 45 min at 65 ÅãC. Radiochemical purity was determined by ITLC. In vitro stability of both radiolabeled species was assessed in saline and serum. In vitro cell binding experiments were performed on integrin ανβ3 receptor-positive U87MG cancer cells. Non-specific Au@DTDTPA was used for comparison. Ex vivo biodistribution studies and in vivo PET and MRI imaging studies in U87MG tumor-bearing SCID mice followed. The Au@DTDTPA nanoparticles were labeled with Gallium-68 at high radiochemical yield (>95%) and were stable at RT, and in the presence of serum, for up to 3 h. The cell binding assay on U87MG glioma cells proved that 68Ga-cRGD-Au@DTDTPA had specific recognition for these cells. Biodistribution studies in U87MG tumor-bearing SCID mice showed that the tumor to muscle ratio increased from 1 to 2 h p.i. (3,71 ± 0.22 and 4,69 ± 0.09 respectively), showing a clear differentiation between the affected and the non-affected tissue. The acquired PET and MRI images were in accordance to the ex vivo biodistribution results. The preliminary results of this study warrant the need for further development of Au@DTDTPA nanoparticles radiolabeled with Ga-68, as possible dual-modality PET/MRI imaging agents.

